# Editorial for Special Issue on Gene Therapy of Rare Diseases

**DOI:** 10.3390/cells15060504

**Published:** 2026-03-12

**Authors:** Cord Brakebusch

**Affiliations:** Biotech Research and Innovation Center (BRIC), University of Copenhagen, Ole Maaløes Vej 5, 2200 Copenhagen, Denmark; cord.brakebusch@bric.ku.dk

## 1. What Are Rare Diseases?

A rare disease is a condition that affects only a small portion of the population. In Europe, rare diseases are defined as diseases with a frequency of less than 1 in 2000. However, more than 7000 rare diseases have been described to date, and although each single disease is rare, the total number of patients suffering from rare diseases is high. It is estimated that 6% of the world population (around 450 million people) are affected by rare diseases [1]. Most rare diseases have a genetic background. Importantly, nearly all rare diseases lack an approved treatment; hence, the development of novel therapies for rare diseases is a major strategic research goal in the European Union, the US, and other countries.

## 2. Challenges for the Treatment of Rare Diseases

Developing therapies for rare diseases is challenging [2]. Gene therapy is the only curative option for the treatment of most rare diseases, but here, many obstacles need to be overcome in the development of an effective drug [3]. Firstly, many different genes and within each gene often different disease-causing mutations need to be targeted. This requires the development of a large number of therapies. Second, since each new therapy addresses only a very small number of patients, the costs per treatment are very high, often exceeding the financial capacities of individual healthcare systems. Gene therapy products such as Lenmeldy, Hemgenix, Skysona, Zynteglo, or Zolgensma, developed for the metachromatic leukodystrophy, hemophilia B, β-thalassemia, cerebral adrenoleukodystrophy, and spinal muscular atrophy, respectively, show clinical efficacy, but with prices of up to $4.3 million per patient, sales are still slow [4]. Moreover, unexpected and expensive failures at stage 3 clinical trials can occur despite convincing animal studies [5]. Finally, gene therapy is associated with several safety issues, including unwanted genetic alterations and immune reactions against the viral vectors used to introduce gene therapy tools into the target cells. More research is therefore required to reduce costs and minimize the adverse effects of gene therapy.

## 3. Content of This Special Issue

This Special Issue on “Gene Therapy of Rare Disease” collects several reviews and one original article covering the ongoing research into increasing the viability of gene therapy as a clinical treatment option.

For recessive rare diseases, where a functional gene is lacking, gene addition therapy—that is, is the introduction of a normal version of the diseased gene into the target cells—is possible. Lin et al. [Contribution 1], in an original article, report the successful gene therapy of a lysosomal storage disorder involving the intracranial viral transduction of the galactocerebrosidase gene into Twitcher mice, a mouse model for globoid cell leukodystrophy. The transduced gene was shown to be widely expressed in both the central and peripheral nervous systems and to greatly improve the survival of the treated Twitcher mice.

Next, Giometti and Papanikolaou [Contribution 2] provide an overview of the currently used viral transduction systems for hematopoietic stem cell gene therapy. This includes improvements to the viral vectors, small molecules enhancing transduction efficiency, safety aspects, ongoing clinical trials, and corresponding gene therapies already on the market.

Crucial for the success of gene addition therapies is the expression of the added gene being sufficiently high and restricted to the target cells. The choice of the promoter directing the expression level and cell type specificity of the added gene is therefore of the utmost importance. Artemyev et al. [Contribution 3] provide a detailed introduction to liver-specific promoters for application in the treatment of rare diseases of the liver, including α1-antitrypsin disease, Wilson disease, and hemophilia A and B. They describe the advantages and shortcomings of all the promoters currently used in liver-directed gene therapy in great detail. In an additional review, Artemyev et al. provide an overview of the attempts to generate synthetic promoters combining high tissue-specificity with high expression levels, thus widening the repertoire of natural promoters currently being used [Contribution 4].

More elegant than therapy by gene addition is therapy by gene repair, which, ideally, changes the defective gene back to the normal variant of the gene. In recent years, CRISPR technologies, including classical CRISPR/Cas9 genome editing, base editing, and prime editing, have given this field a significant boost. In 2025, Casgevy, a CRISPR-based cure for sickle cell anemia and transfusion-dependent β-thalassemia, was approved by the FDA, and more than 100 clinical trials for CRISPR-based medicines are ongoing. Mentani et al. [Contribution 5] describe the molecular mechanisms underlying the repair of DNA double-strand breaks, as well as the prime editing technology, which requires only a single-strand DNA break, resulting in less off-target effects.

Improving the efficiency and specificity of gene editing is a major research goal in CRISPR gene editing. He and Brakebusch [Contribution 6] discuss the question of whether modulation of nuclear actin polymerization could be used to increase the efficiency of precise CRISPR/Cas9 genome editing by homologous recombination.

Finally, two reviews present the current state of the art in gene therapy for specific rare diseases. Leal et al. describe the challenges and opportunities encountered in treating lysosomal storage disorders via CRISPR/Cas technologies [Contribution 7], and Vale et al. demonstrate the gene therapy options for the treatment of Diamond-Blackfan anemia [Contribution 8].

## 4. Conclusions

Taken together, gene therapy for rare diseases is a vibrant research field that, increasingly, is producing drugs approved for clinical application. However, the costs of these curative therapies need to be reduced in order to promote their wider application.

## Data Availability

No new data were created or analyzed in this study. Data sharing is not applicable to this article.
